# Serum BDNF and Selenium Levels in Elite Athletes Exposed to Blows

**DOI:** 10.3390/medicina58050608

**Published:** 2022-04-27

**Authors:** Murat Ozan, Yusuf Buzdağli, Nurcan Kılıç Baygutalp, Neslihan Yüce, Fatih Baygutalp, Ebubekir Bakan

**Affiliations:** 1 Department of Physical Education and Sports, Kazım Karabekir Education Faculty, Ataturk University, Erzurum 25240, Turkey; murat.ozan@atauni.edu.tr; 2 Department of Physical Education and Sports, Faculty of Sport Sciences, Erzurum Technical University, Erzurum 25240, Turkey; yusuf.buzdagli@erzurum.edu.tr; 3 Department of Biochemistry, Faculty of Pharmacy, Ataturk University, Erzurum 25240, Turkey; 4 Department of Medical Biochemistry, Faculty of Medicine, Ataturk University, Erzurum 25240, Turkey; neslihan.yuce@atauni.edu.tr; 5 Department of Physical Medicine and Rehabilitation, Faculty of Medicine, Ataturk University, Erzurum 25240, Turkey; fatihbaygutalp@atauni.edu.tr; 6 Department of Biochemistry, Faculty of Medicine, Ağrı İbrahim Çeçen University, Ağrı 04100, Turkey; ebakan@atauni.edu.tr

**Keywords:** BDNF, blows, exercise, selenium

## Abstract

*Background and Objectives*: The study aimed to investigate the combined acute and long-term effects of exposure to blows and exercise on serum BDNF (brain-derived neurotrophic factor) and selenium levels. *Materials and Methods*: Serum BDNF and selenium levels were determined in 40 male elite athletes before and after vigorous exercise (training match) with a probability of exposure to blows and in 10 sedentary men subjected to exercise (Astrand running protocol). *Results*: Serum BDNF levels were found 11.50 ± 3.50 ng/mL before exercise and 14.02 ± 3.15 ng/mL after exercise in the athlete group (*p* = 0.02), and 12.18 ± 4.55 ng/ mL and 11.74 ± 2.48 ng/ mL before and after exercise in the sedentary group, respectively (*p* = 0.873). Serum BDNF (pre-exercise, baseline) levels were slightly lower in the athlete group than those in the sedentary group (11.50 ± 3.50 and 12.18 ± 4.55 ng/mL, respectively, *p* = 0.796). Pre-exercise serum selenium levels in athletes were significantly higher compared to those of sedentary participants (130.53 ± 36.79 and 95.51 ± 20.57 µg/L, respectively, *p* = 0.011). There was no difference in selenium levels after exercise (124.01 ± 29.96 µg/L) compared to pre-exercise (130.53 ± 36.79 µg/L) in the athlete group (*p* = 0.386). Similarly, there was no difference in selenium levels after exercise (113.28 ± 25.51 µg/L) compared to pre-exercise (95.51 ± 20.57 µg/L) in the sedentary group (*p* = 0.251). *Conclusions*: BDNF results show that even if athletes are exposed to blows, they may be protected from the long-term effects of blows thanks to the protective effect of their non-sedentary lifestyle. Regular exercise may have a protective effect on maintaining serum selenium levels in athletes even exposed to blows chronically.

## 1. Introduction

BDNF (brain-derived neurotrophic factor) is a protein that affects neuronal survival, neurogenesis, and neuroplasticity in the central nervous system and is involved in cell differentiation, axon and dendrite growth, synapse formation, and synaptic plasticity. Since the expression of BDNF is dependent on neuronal activity, BDNF mainly helps neuronal development and renews in the central nervous system and contributes to the maintenance of important neuronal pathways. Therefore, BDNF applications have gained importance in the treatment of neurodegenerative diseases. BDNF expression and concentration vary in physiological and pathological conditions [1]. Previous studies have also shown that basal BDNF levels are lower in individuals who do not exercise or are not active in any sports types [2,3,4]. At the same time, serum BDNF levels were reported higher in athletes in combat sports after exercise compared to other sports types [5]. Studies reporting that exercise has cognitive benefits suggest that BDNF is involved in this mechanism [6,7,8]. Acute exercise has dynamic effects on memory functions [9,10,11]. Exercise type and duration play a role in the effects of exercise on cognitive functions. In a comprehensive review, Yamada et al. hypothesized that exercise that restricts blood flow could improve several cognitive domains such as attention, executive function, and memory [12].

There are a few studies on the effect of BDNF on the performances of athletes, which report different results. Kim et al. reported that the basal BDNF level of the athletic adolescents, who were regular athletes, was lower than the control group [13]. However, it was found that aerobic or anaerobic acute training increased serum BDNF levels in both the control group and athletes [14]. It has been determined that a 12-week resistance training exercise program applied to healthy individuals did not cause an increase in basal BDNF concentrations [15]. This may be attributed to the exercise type because aerobic exercise caused an increase in basal BDNF concentrations. Again this result should be confirmed with further studies in different populations. In a study examining the effects of different environmental factors on performance, it was seen that exercise increased BDNF significantly, but the altitude had no effect on the BDNF concentration [16]. In recent studies, it has been observed that, contrary to the above studies, exercise increases the serum level of BDNF, and even different types of exercise can affect the level of this increase. It has been reported that the increase in BDNF levels varies in different types of training [6]. In a different study, an increase in peripheral serum BDNF was reported with high-intensity exercise [16]. While high-intensity interval training (HIIT) supports the view that it provides higher fat oxidation in skeletal muscle compared to other forms of exercise, significant increases in BDNF serum levels have also been found [17]. It has been reported that high-intensity exercise performed at short intervals is more effective than continuous high-intensity exercise for increasing serum BDNF [18]. Additionally, some studies have reported that both acute and chronic exercise increases serum BDNF concentrations [19]. Although there was an increase in BDNF concentration within 30 min after exercise with acute exercise, it was observed that there was no increase in basal level when evaluated as chronic [20].

It is well known that several elements play a role as cofactors in many enzyme actions in our body. Body stores of various trace elements are affected by physical exercise, and their redistribution between blood and tissues occurs, indicating a possible relationship between sportive performance and element metabolism. For this reason, it may be important to show how exercise affects the element distribution within body compartments. Of these elements, selenium has biological activity identified as selenoproteins [21,22]. Glutathione peroxidase, a selenium-dependent enzyme, is the most important component of the body’s antioxidant system.

Some sport types; for example, boxing, taekwondo, and soccer include exposure to blows and a high risk of direct injury. The effects of repeated blows can be seen in the following years in athletes who do these types of sports. This study aims to investigate the acute effects of vigorous exercise (a training match) on serum BDNF and selenium levels in athletes from different sports types involving the possibility of exposure to blows and to evaluate the relationships between the two parameters. The evaluation of the chronic effects of exercise on serum BDNF and selenium levels is another aim of the study by considering the pre-exercise (baseline) values of athlete groups and sedentary individuals. In addition, the study may reach an understanding of whether serum BDNF levels are a candidate biomarker for determining exposure levels of blows seen in some traumatic sport types.

## 2. Materials and Methods

In this study, it was requested to meet the criteria of not having any acute or chronic diseases, including neurological disorders, which are included in the combat sports (athletes exposed to blows), at least eight years of age in the athlete group.

Forty male elite athletes, including boxing (n:10), taekwondo (n:10), wrestling (n:10), and soccer (n:10), and ten sedentary men were included in the study as a control group. No participants had selenium supplements. Venous blood samples were taken before and after training from all participants, with the total specimen being 100 samples. No participants have health problems.

The subjects’ demographic data included ages, sports ages of the athletes, and height (by a portable height gauge) were collected (Seca 216, Hamburg/Germany, 2020).

### 2.1. Exercise Protocols

#### 2.1.1. For Wrestling, Boxing, and Taekwondo

Elite athletes joining in international competitions and having a sports age of at least 8 years were included in the study. The competition model was applied to the athletes in their own weight in accordance with the international competition rules. All athletes warmed up for 20 min before the competition. Then they were subjected to training matches: in wrestling competition consisting of 2 periods for 2 × 3 min (30 s rest between halves), in boxing competition consisting of 3 rounds for 3 × 3 min (1-min rest between rounds); and in taekwondo competition consisting of 3 rounds for 3 × 2 min (1-min rest between rounds).

#### 2.1.2. For Football Players

Ten elite football players from professional football teams with a sports age of at least 8 years participated in the study. In accordance with the international competition rules, the athletes were given a training match equivalent to the competition consisting of 2 halves (15 min rest between half-time) for 2 × 45 min.

#### 2.1.3. For Sedentary Participants

The Astrand running protocol was used as an exercise protocol for sedentary participants. Accordingly, sedentary individuals started the warm-up period at a speed of 7 km/h and performed the warm-up runs at the determined speed within 5 min, and 1 min of active rest was given. With the completion of the warm-up run, a constant speed of 8.05 km/h was used in the exercise protocol. The slope was increased to 2.5% after the participants ran at a 0% slope for the first 3 min. Afterward, the slope was increased by 2.5% every 2 min. Individuals continued to exercise until they were exhausted. After the exercise protocol was completed, the speed was reduced to 4 km/h and a 5-min cooling run was performed.

### 2.2. Body Composition Measurements

The participant’s body composition measurements were made by a composition device (Tanita TBF 300/Arlington Heights, IL, USA, 2010) while dressed in shorts and t-shirts, in bare feet and anatomical posture, and with a precision of 0.1 kg.

### 2.3. Specimen Collection and Biochemical Analyses

Venous blood samples were taken from athletes and the control group immediately before and about 10 min after the exercise program. Participants were not allowed to take drugs, caffeine, alcohol, and performance-enhancing ergogenic supplements and exercise until 48 h before the study. During the study schedule, participants were also told to avoid foods and beverages containing selenium.

Venous blood samples of the control group will be taken in a sitting position. Dry tubes with gel separators (Vacuette, Greiner Bio-one GmbH, Kremsmünster, Austria) containing a clot activator will be used to obtain serum. The blood samples will be centrifuged at 1250× *g* for 10 min in the Medical Biochemistry laboratory of Atatürk University Faculty of Medicine and the serum will be separated. After centrifugation, the serum samples were aliquoted and stored in a freezer (HERA Freeze, Thermo Fisher Scientific, Waltham, MA, USA) at −80 °C. until the analysis day.

Serum BDNF levels were analyzed by the ELISA method (YL Biont, Catalog no: YLA0580HU) with a commercial kit. The % CV values of the test are reported by the manufacturer as ±10%, with a range of 0.05–10 ng/mL. This kit, which works with the sandwich method, has a sensitivity of 0.01 ng/L. Results are given as ng/mL for serum samples.

For selenium measurements, 1 mL of serum sample was burned in a microwave oven (MILESTONE Ethos Easy Advanced Microwave Digestion System) with 5 mL of supra pure nitric acid (HNO3¯) and 5 mL of deionized water in Teflon containers (Milestone D5, Lake Oswego, OR, USA). After cooling, the burned samples were transferred to 50 mL polypropylene tubes and diluted to 20 mL with deionized water. Serum selenium concentration measurements were performed using the Agilent ICP-MS system (7700 series ICP-MS, Agilent Technologies, Santa Clara, CA, USA). The limit of detection (LOD) values for Se was 0:018 lg = L. For Se, the between-assay coefficients of variation (CVs) were 0.36% and the within-assay CVs were 0.89%.

### 2.4. Statistical Analyzes

Statistical analyzes were performed using the SPSS 23.0 package program. The Kolmogorov-Smirnov test was used to evaluate the normality of data. Descriptive statistical analyzes, dependent groups *t*-tests, and independent groups *t*-tests were performed. Values of *p* < 0.05 at a 95% confidence interval were considered statistically significant.

## 3. Results

The demographical characteristics of the participants are given in Table 1. Athletes and sedentary participants were well matched in terms of age, body mass index (BMI), and body fat percentage (BFP) (*p* > 0.05 for all comparisons). There was a significant difference in basal metabolic rate (BMR) between athletes and sedentary participants (*p* = 0.012).

**Figure 1:**
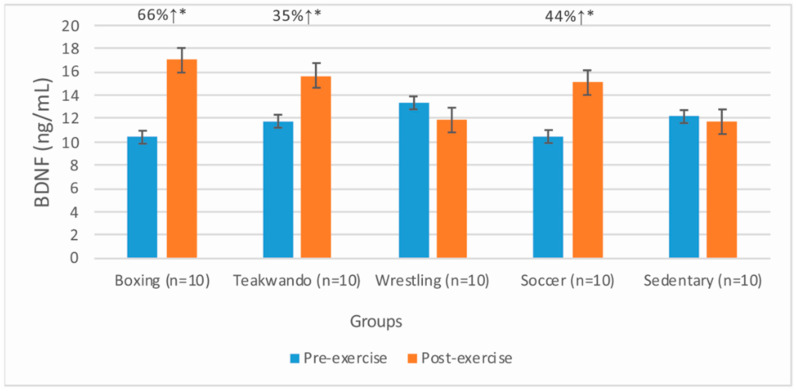
Alterations in serum BDNF levels induced by training in different sport types and sedentary participants. ↑: increment. *: % alteration is significant at *p* < 0.05 level.

Acute effects of training on serum BDNF levels are given in Table 2. Alterations (as %) in serum BDNF levels in different sport types and sedentary participants are given in Figure 1. The post-exercise serum BDNF levels were significantly higher compared to pre-exercise values for athletes (*p* = 0.002).

Long-term effects of exercise and exposure to blows on serum BDNF and selenium levels are summarized in Table 3. The pre-exercise values of serum selenium levels in athletes (baseline values) were significantly higher compared to pre-exercise values of sedentary participants (*p* = 0.011).

## 4. Discussion

Boxing and other combat sports are known to expose participants to severe blows [20], resulting in headaches, depression, memory problems, and gait disturbances. Professional athletes take the blows to their head, face, and neck. The future, long-term neurological consequences of cumulative blows include walking difficulties, Parkinson’s disease, and Alzheimer’s disease [23], which are infrequently seen in athletes engaged in amateur combat [24].

Traumatic brain injury has been shown to reduce serum BDNF levels and its expression in tissues [25]. In the study conducted by Oztasyonar, serum BDNF values were measured before and after training in three groups of athletes and sedentary individuals. Pre-training, basal serum BDNF values of all athletes were higher than sedentary individuals, and serum BDNF levels increased after training [26]. It is known that serum BDNF levels increase with the effect of exercise/training in martial athletes by the beneficial effects of exercise, and BDNF levels are expected to decrease with the effect of the blows in martial athletes [5]. However, studies in the literature report that serum BDNF levels increase as a protective action of exercise in these athletes immediately after training, which probably helps maintain the brain function within physiological ranges. Consequently, exercise has not only metabolic but also cognitive benefits, and determining the net effect of acute and long-term exercise on serum BDNF levels in these athletes constitutes an important purpose of our study. There are limited studies with controversial results about BDNF secretion by the effects of blows and the relationship between BDNF and exercise in the literature due to the differences in research methodologies (aerobic/anaerobic exercise type, exercise intensity, exercise duration, study population age, gender. etc.).

Our results showed that there was no significant alteration in wrestling athletes in terms of BDNF levels after training. There is one available study in the literature with a somewhat similar methodology evaluating serum BDNF levels in wrestling athletes. In that study, serum BDNF levels were increased after a fighting match (high intensity, duration 3 × 2 min) in an athlete group including judo, sumo, and wrestling [27]. In our study, wrestling athletes were submitted to a competition consisting of 2 periods for 2 × 3 min. The different results of the two studies may arise from different methods (competition periods) and biological variations arising from different populations.

In this study, serum BDNF levels were measured before and after vigorous exercise (training match) in four different groups of elite athletes with a high probability of exposure to blows and in sedentary individuals before and after exercise. We hypothesized that there are two factors affecting serum BDNF levels. Exposure to blows may decrease BDNF levels and a non-sedentary lifestyle may increase BDNF levels. It was thought that the experimental group subjected to the study was exposed to blows for many years due to the sports type and this situation would have an effect on the pre-values. Post values were recorded immediately after the competition, which shows the acute effects of blows. Pre- and post-exercise BDNF values in athlete groups were compared to evaluate the acute effects of exposure to blows, and whether there will be a decrease in BDNF levels. Pre-exercise values of athletes and sedentary subjects were compared to evaluate the long-term effects of exposure to blows because athletes had a sports age of approximately eight years. The comparison of pre- and post-exercise BDNF values in athlete groups showed increased levels, which is an indication of the more increasing effect of exercise on BDNF than the decreasing effect of blows on BDNF, a kind of compensation of training for the effects of blows thanks to the positive effects of training. In other words, even if the athletes are exposed to acute blows, the protective effect of their non-sedentary lifestyle can protect them from the long-term effects of repetitive blows.

On the other hand, comparing the pre-exercise (baseline) BDNF values of athletes and sedentary individuals showed that the long-term effects of blows in athletes slightly, insignificant decreased serum BDNF levels. The possible effects of long-term repetitive blows on decreasing serum BDNF levels should therefore be investigated in detail. Further studies are needed with a larger sample size, different types, and different exercise protocols in order to provide more information about the relationship between BDNF and sports performance. Our results will provide important data for future studies that will investigate the relationship between sportive performance and BDNF.

There is a limited number of studies investigating the effects of exercise on serum selenium levels. In a study, serum selenium levels determined by ICP-MS revealed that baseline selenium levels were significantly lower in athletes than in sedentary controls [5]. Maynar et al. measured serum and urinary selenium levels before and after an acute physical activity until exhaustion in 21 middle- and long-distance runners and 26 sedentary participants [28]. Authors have reported a decrement in selenium levels after performing an acute physical activity until exhaustion [28]. This decrement may arise from the exercise protocol which was performed until exhaustion [29]. Increased oxidative stress activates antioxidant enzymes and this will lead to a decrement in selenium levels [30]. Experimental animal studies show that selenium levels dramatically decrease after trauma, which may be associated with multiple organ failures following severe trauma [31]. Seelig et al. measured serum selenium levels in fifty-two traumatic spinal cord injury patients. Researchers reported a strong relationship between temporal changes in the selenium status and the prognosis after traumatic spinal cord injury. Furthermore, they concluded that selenium levels might be used as a potential predictor for the remission of traumatic spinal cord injury [32].

Hypothetically, athletes are assumed to have higher antioxidant status compared to sedentary people, and they represent a model of high antioxidant status in observational studies due to their chronic adaptation to oxidative stress [33]. Our results have shown that baseline serum selenium levels were higher in athletes compared to sedentary people, thanks to their adaptation to oxidative stress conditions. In addition, athletes have a regular nutrition program, avoiding fast foods. These factors may lead to higher baseline selenium levels in athletes compared to sedentary people.

Our participants in the athlete group consisted of athletes in boxing, taekwondo, wrestling, and football. Severe injury may occur, especially in combat sports. Therefore, selenium levels should be maintained in athletes involved in combat sports and athletes, particularly exercising until exhaustion. Additionally, these results emphasize that intense exercise has negative consequences on metabolism.

The relatively small sample size of the study is a limitation of the study. This situation was due to the issues related to the COVID-19 pandemic (decrease in sporting activities and concerns about giving blood sampling).

## 5. Conclusions

Even if athletes are exposed to blows, as in some sporting types, they may be protected from the long-term effects of blows thanks to the protective effect of their non-sedentary lifestyle, shown by BDNF results. We could not reach the aim to show BDNF as a biomarker for exposure to blows, because there is a confounding effect of chronic exercise in the athlete population. Regular exercise may have a protective effect on maintaining serum selenium levels in athletes even exposed to blows chronically. Further studies in larger groups including different populations are needed to investigate BDNF as a candidate biomarker, particularly in long-term exposure to blows.

## Figures and Tables

**Table 1:** Demographical characteristics of participants.

	Athletes (*n* = 40), Male	Sedentary (*n* = 10), Male	*p*
Age (year)	21.6 ± 3.0	19.8 ± 1.3	0.067
BMI (kg/m^2^)	23.9 ± 3.3	22.1 ± 1.9	0.107
BMR (kcal)	1809.3 ± 169.0	1700.8 ± 95.4	0.012
BFP	8.8 ± 4.6	7.3 ± 3.9	0.297

*p*: independent samples *t*-test statistics *p*-value, BMI: Body mass index, BMR: Basal metabolic rate, BFP: body fat percentage.

**Table 2:** Acute effects of training on serum brain-derived neurotrophic factor (BDNF) and selenium levels.

Serum BDNF Values (ng/mL)			
	Pre-Exercise	Post-Exercise	*p*
Athletes (*n* = 40)	11.50 ± 3.50	14.02 ± 3.15	0.002
Sedentary (*n* = 10)	12.18 ± 4.55	11.74 ± 2.48	0.873
**Serum Selenium Values (µg/L)**			
	**Pre-Exercise**	**Post-Exercise**	***p***
Athletes (*n* = 40)	130.53 ± 36.79	124.01 ± 29.96	0.386
Sedentary (*n* = 10)	95.51 ± 20.57	113.28 ± 25.51	0.251

*p*: dependent samples *t*-test statistics *p*-value.

**Table 3:** Long-term effects of exercise and exposure to blows on serum BDNF and selenium levels.

	Serum BDNF Values (ng/mL)	
	Pre-Exercise	*p*
Athletes (*n* = 40)	11.50 ± 3.50	0.796
Sedentary (*n* = 10)	12.18 ± 4.55	
	**Serum Selenium Values [µg/L]**	
	**Pre-Exercise**	***p***
Athletes (*n* = 40)	130.53 ± 36.79	0.011
Sedentary (*n* = 10)	95.51 ± 20.57	

*p*: independent samples *t*-test statistics *p*-value.

## Data Availability

The datasets generated during and analyzed during the current study are available from the corresponding author on reasonable request.

## Previous Presentation

This study was partly presented in 31th National Biochemistry Congress (Turkey) on 19 December 2020.
